# Long-term care hospitals as end-of-life care settings in South Korea: A nationwide analysis of utilization patterns and clinical trajectories

**DOI:** 10.1371/journal.pone.0354271

**Published:** 2026-07-24

**Authors:** Haein Kim, Seungwon Shin

**Affiliations:** College of Korean Medicine, Sangji University, Wonju, Republic of Korea; Taipei Medical University, TAIWAN

## Abstract

**Objectives:**

This study aimed to investigate the utilization patterns and end-of-life trajectories of older adults in Korean long-term care hospitals (LTCHs) using nationwide population-based data.

**Methods:**

This retrospective observational study analyzed National Health Insurance Service claims data (2014–2023). We constructed three analytical groups: an annual inpatient group to examine longitudinal trends in volume and mortality; a newly admitted inpatient group to assess the sociodemographic profile at the time of LTCH entry; and a decedent group to reconstruct the clinical trajectory and cumulative resource use from initial admission to death.

**Results:**

While the volume of older inpatients increased, the annual number of deaths involving LTCH use increased from 68,357 in 2014 to 112,157 in 2023 over the decade. Patients were newly admitted to LTCHs in their early 80s (median age 81 years, IQR 75–86) and were predominantly female (62.1%). In addition, Medical Aid beneficiaries (13.7%) and individuals with registered disabilities (28.0%) were overrepresented, indicating high sociodemographic vulnerability. Decedents spent a median of 79 days (IQR 22–299) as LTCH inpatients. While 30.1% stayed for <30 days, 22.2% remained hospitalized for ≥ 360 days, indicating substantial variation in length of stay. Overall, 68.5% of decedents died during LTCH stay. While admissions were driven by chronic geriatric syndromes, the end-of-life phase was dominated by a surge in acute infectious and systemic conditions (e.g., pneumonia, 10.2% to 22.3%; sepsis, 3.3% to 11.9%).

**Conclusions:**

LTCHs in Korea have played an increasingly important role as a setting for end-of-life care, managing a distinct trajectory from chronic frailty to acute deterioration. Given that these institutions are functioning as end-of-life care settings, policy reforms should strengthen palliative care integration, advance care planning, workforce training, and end-of-life care capacity within LTCHs.

## Introduction

Population aging is accelerating worldwide [1], and South Korea (hereafter, Korea) is undergoing an exceptionally rapid demographic transition [2]. Older adults frequently present with multimorbidity and functional decline, driving the demand for long-term care [3]. Healthcare utilization among older populations tends to concentrate near the end of life [4], making geriatric care a critical health policy challenge in Korea [5].

Korea operates a dual long-term care system in which long-term care hospitals (LTCHs) and nursing homes are independently financed and governed under the National Health Insurance (NHI) and Long-Term Care Insurance, respectively [6]. Under the Medical Service Act, LTCHs are physician-staffed, hospital-level medical institutions that primarily provide inpatient clinical care, ongoing medical management, and rehabilitation [7]. In contrast, nursing homes are welfare facilities under the Welfare of Older Persons Act, focusing on assistance with activities of daily living and basic nursing care [8].

In Korea, deaths among older adults increasingly occur in hospitals and other institutional settings rather than at home [9]. Although LTCHs were established primarily for subacute and rehabilitative care, they may be playing an increasingly important role in this institutional end-of-life care. The divergence between relatively stable LTCH admissions and a disproportionate rise in LTCH-associated deaths suggests an evolving role that has not been comprehensively investigated at the national level. Although previous studies on Korean LTCHs have investigated specific clinical outcomes (e.g., length of stay [LOS] or functional status), they often lacked a comprehensive evaluation of longitudinal end-of-life trajectories and diagnostic shifts that could explain this changing role [10–16]. Consequently, nationally representative data detailing the overall scale of LTCH utilization, patient sociodemographics, and mortality patterns are lacking. Also, comprehensive evidence regarding clinical trajectories, including shifts in major diagnoses from admission to the end of life, remains scarce.

This study aimed to investigate the utilization patterns and end-of-life trajectories of older adults (≥ 65 years) admitted to Korean LTCHs, using nationwide population-based claims data from the National Health Insurance Service (NHIS) between 2014 and 2023. Specifically, we identified annual trends in admissions and mortality, assessed cumulative healthcare utilization, and compared major diagnoses at LTCH entry versus those at the final claim before death to delineate end-of-life clinical trajectories. We anticipated that LTCHs in Korea would increasingly serve as primary end-of-life care settings, managing high mortality burdens and distinct clinical trajectories beyond their traditional role in subacute rehabilitation.

## Materials and methods

The study was conducted in accordance with the ethical standards of the Helsinki Declaration and was exempt from review by the Institutional Review Board of Sangji University (Wonju, Republic of Korea; No. 2024−15). The review board waived the requirement for informed consent because this study used anonymized administrative data. The authors had no access to information that could identify individual participants during or after data collection. Research data was accessed on July 1, 2025. This manuscript was prepared following the Strengthening the Reporting of Observational Studies in Epidemiology (STROBE) reporting guideline [17].

### Study design and data sources

This nationwide, retrospective, observational study used whole-population claims data from the NHIS of Korea. Under Korea’s mandatory single-payer system, which covers approximately 97% of the population [18], comprehensive claims data across all medical institutions are captured and provided in de-identified format. This longitudinal database integrates records on demographics, diagnoses, prescriptions, medical procedures, and institutional characteristics, alongside mortality data from Statistics Korea [19].

### Study population

We sequentially constructed three distinct analytical groups from the whole-population claims database, encompassing all LTCH admissions between 2014 and 2023. Individuals with missing or invalid data for key variables were excluded across all stages of study group construction:

Annual inpatient group (2014–2023): Constructed to explore the annual volume and trends of older LTCH inpatients, this group included all individuals aged ≥ 65 years who had at least one LTCH inpatient claim during a given calendar year.

Newly admitted inpatient group (2015–2023): Built to evaluate sociodemographic profile at the time of LTCH entry, this group included individuals aged ≥ 65 years whose first observed LTCH claim occurred between 2015 and 2023, strictly excluding those with any prior LTCH admission history recorded in the dataset since 2014 using a minimum one-year washout period based on available claims data. Because claims data prior to 2014 were unavailable, a longer washout period could not be applied.

Decedent group (2015–2023): Designed to assess cumulative healthcare utilization (LOS and medical costs) and diagnostic shifts from admission to the end of life, this group comprised individuals from the incident group whose deaths were confirmed during the study period.

### Study measures and operational definitions

#### Annual volume and mortality of LTCH patients.

Annual older LTCH users were defined as individuals aged ≥ 65 years with at least one LTCH inpatient claim during a given calendar year. New admissions were identified as the first observed LTCH inpatient claim between 2015 and 2023, excluding those with any prior admissions recorded since 2014 (a minimum one-year washout period). Annual decedents were identified by linking the NHIS database to Statistics Korea data. This group comprised individuals who died in a given year and had at least one LTCH claim during that same year, reflecting mortality specifically among the LTCH user population.

#### Sociodemographic profile at admission.

Sociodemographic characteristics were assessed at the time of first admission in the Newly admitted inpatient group. Age was calculated by subtracting the birth year from the year of admission. Region of residence was categorized into the capital area (Seoul, Incheon, Gyeonggi), non-capital metropolitan cities (Busan, Daegu, Gwangju, Daejeon, Ulsan), and other regions. Public health insurance status was classified as either NHI or Medical Aid. Disability status was classified as present or absent based on the national disability registry.

#### Cumulative healthcare utilization and end-of-life trajectories.

The cumulative LOS and total medical costs were calculated by summing all inpatient days and medical expenses from the first LTCH admission through to death. Total medical costs were defined as the aggregate of the insurer’s reimbursements and patient co-payments for services covered by the NHI. Time from admission to death was calculated as the number of days between the initial LTCH admission date and the date of death. To account for potential administrative billing discrepancies, negative time values from admission to death were recoded as zero. Death during LTCH stay was defined as when the date of death coincided with the calculated discharge date of the final LTCH admission (admission date + LOS – 1 day).

#### Changes in recorded clinical conditions.

To map clinical trajectories, we identified all primary and secondary International Classification of Diseases, Tenth Revision (ICD-10) codes recorded on the date of the first admission and the final claim preceding death. Codes from multiple claims on the same date were aggregated to capture the full diagnostic profile. Diseases were categorized according to the HIRA classification guidelines [20], with additional high-frequency diagnoses reported separately if not captured by the HIRA grouping criteria.

### Statistical analysis

All analyses were descriptive. Continuous variables are presented as medians with interquartile ranges (IQR), while categorical variables are reported as frequencies and percentages. We summarized annual counts and proportions by calendar year and calculated compound annual growth rates (CAGR) based on the first and last observed annual values to quantify overall temporal changes during the study period. In addition, linear regression analyses were performed across the entire study period to assess temporal trends. All analyses were performed using SAS Enterprise Guide 7.1 (SAS Institute Inc., Cary, NC, USA).

To visualize the clinical trajectory from admission to end-of-life, we calculated the percentage change in prevalence for each condition. We mapped these shifts to highlight the transition from chronic morbidities to acute end-of-life conditions. Data visualization was conducted using R software 4.5 (R Foundation for Statistical Computing, Vienna, Austria).

## Results

### Selection of the study population

**Fig 1:**
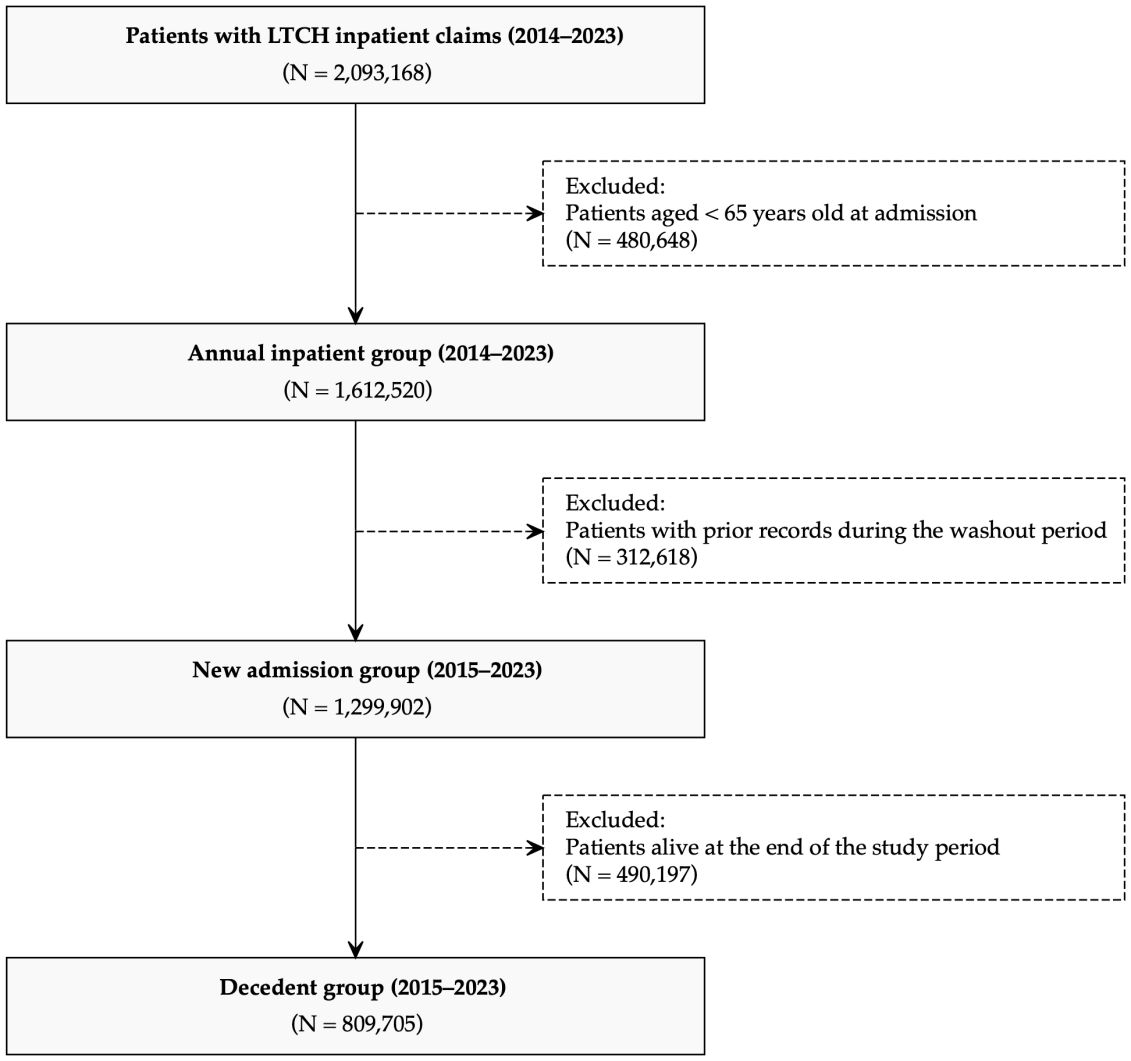
Flowchart of study population selection and study group construction. Abbreviation: LTCH, long-term care hospital. Source: National Health Insurance Service (NHIS) claims data, 2014–2023.

From a total of 2,093,168 individuals with LTCH inpatient claims between 2014 and 2023, we excluded those aged < 65 years, resulting in an annual inpatient group of 1,612,520 unique older adults. After excluding 312,618 patients with prior admissions during the washout period, we identified 1,299,902 individuals for the newly admitted inpatient group (2015–2023). Within this group, 809,705 individuals were confirmed to have died during the study period, forming the decedent group. Fig 1 illustrates the construction of the three analytical groups.

### Annual trends in patient volume and mortality

Between 2014 and 2023, the annual number of older individuals with at least one LTCH inpatient claim grew from 278,858 to 360,948 (CAGR 2.91%). While there was a transient decline during the COVID-19 period (2019–2020), the volume subsequently recovered. The proportion of older adults among the total inpatient population (all ages) rose by 5.0 percentage points, from 77.7% in 2014 to 82.7% in 2023. Within this annual group, the volume of newly admitted patients increased from 141,524 in 2015 to 151,688 in 2023 (CAGR 0.87%), showing a temporary decrease in 2020 followed by a rebound. The proportion of older adults among all new admissions increased by 4.7 percentage points (from 76.7% to 81.4%) over the same period. Regarding mortality, the annual number of older decedents rose sharply from 68,357 in 2014 to 112,157 in 2023 (CAGR 5.66%). Older adults accounted for an increasing proportion of all deaths via LTCHs, rising from 89.2% in 2014 to 91.4% in 2023.

Table 1 presents the annual figures for the older inpatient group, including their proportion relative to the total population. The corresponding statistics for the total LTCH population (all ages), which served as the denominator for these proportions, are provided in S1 Table.

**Table 1:** Annual trends in older inpatients, new admissions, and deaths in LTCHs, 2014–2023 (annual inpatient group, N = 1,612,520).

Year	Total inpatients	New admissions	Deaths
2014	278,858 (77.7)	NA	68,357 (89.2)
2015	302,850 (78.8)	141,524 (76.7)	75,012 (89.7)
2016	324,480 (79.2)	142,697 (76.8)	78,776 (90.0)
2017	347,664 (79.7)	147,447 (77.0)	85,956 (90.4)
2018	366,603 (79.1)	151,878 (75.4)	92,587 (90.6)
2019	372,026 (77.9)	148,965 (74.3)	93,512 (90.1)
2020	347,023 (78.1)	124,409 (74.5)	93,055 (90.3)
2021	343,129 (79.7)	129,353 (77.1)	94,682 (90.4)
2022	382,918 (82.4)	161,941 (80.7)	120,017 (91.6)
2023	360,948 (82.7)	151,688 (81.4)	112,157 (91.4)
**CAGR (%)**	**2.91**	**0.87**	**5.66**
**p for trend**	**0.006**	**0.727**	**< 0.001**

Abbreviations: CAGR, compound annual growth rate; LTCH, long-term care hospital; NA, not applicable.

Data are presented as numbers (%). Percentages in parentheses represent the proportion of older adults (aged ≥ 65 years) relative to the total number of individuals (all ages) within each category. New admissions data for 2014 are not available due to the washout period applied to define incident cases. CAGR was calculated using the first and last observed annual values. P for trend was obtained using linear regression analyses across annual data points.

Source: National Health Insurance Service (NHIS) claims data, 2014–2023.

### Sociodemographic profile at admission

The newly admitted inpatient group (2015–2023) was predominantly female (62.1%), with a median age of 81 years (IQR 75–86). The largest age group comprised those aged 80–84 years (25.4%). Regarding region of residence, the highest proportion of patients resided in non-capital and non-metropolitan areas (Other, 43.6%), followed by the capital area (35.2%). While the majority were covered by NHI (86.3%), 13.7% were Medical Aid beneficiaries, indicating a notable proportion of individuals with low economic status. Sociodemographic characteristics of this group are summarized in Table 2.

**Table 2:** Sociodemographic characteristics of newly admitted older inpatients in LTCHs, 2015–2023 (newly admitted inpatient group, N = 1,299,902).

Characteristic		N (%) or Median (IQR)
Sex	Male	492,669 (37.9)
	Female	807,233 (62.1)
Age at admission (years)		81 (75–86)
	65–69	124,140 (9.6)
	70–74	162,633 (12.5)
	75–79	256,136 (19.7)
	80–84	330,314 (25.4)
	85–89	265,159 (20.4)
	90–94	124,201 (9.6)
	≥ 95	37,319 (2.9)
Region of residence	Capital area	457,965 (35.2)
	Non-capital metropolitan	274,715 (21.1)
	Other	567,222 (43.6)
Public health insurance	Medical Aid	178,084 (13.7)
	NHI	1,121,818 (86.3)
Premium (KRW)		97,490 (23,240–189,890)
Disability	Yes	363,305 (28.0)
	No	936,597 (72.1)

Abbreviations: IQR, interquartile range; KRW, Korean Won; LTCH, long-term care hospital; NHI, National Health Insurance.

Values are presented as numbers (%) or median (IQR).

Source: National Health Insurance Service (NHIS) claims data, 2014–2023.

### Profile of LTCH utilization from admission to death

Analysis of the decedent group revealed a distinct pattern of end-of-life institutionalization. Patients were admitted at a median age of 82 years (IQR 77–87) and died at a median age of 84 years (IQR 79–89). The median interval from initial admission to death was 196 days (IQR 42–762). (The number of cases with negative time values in the calculation of time from admission to death was only 8 cases in the decedent group.) Within this timeframe, the median cumulative LOS was 79 days (IQR 22–299), although utilization patterns were highly variable. While 30.1% stayed for less than 30 days, a large proportion (22.2%) remained hospitalized for over a year (≥ 360 days). Regarding expenditures, the median cumulative medical cost from admission to death was approximately 7.3 million Korean Won (KRW); 42.2% incurred cumulative medical costs of ≤ 5 million KRW, whereas 30.5% incurred costs of ≥ 20 million KRW. Ultimately, 68.5% of the decedents utilized LTCHs as their final point of care at the end of life. Utilization profiles and end-of-life outcomes are presented in Table 3.

**Table 3:** Profile of LTCH utilization from admission to death, 2015–2023 (decedent group, N = 809,705).

Characteristic		N (%) or Median (IQR)
Age at admission (years)		82 (77–87)
	65–69	52,213 (6.5)
	70–74	80,474 (9.9)
	75–79	148,879 (18.4)
	80–84	213,441 (26.4)
	85–89	187,287 (23.1)
	90–94	96,013 (11.9)
	≥ 95	31,398 (3.9)
Age at death (years)		84 (79–89)
	65–69	38,359 (4.7)
	70–74	68,848 (8.5)
	75–79	124,794 (15.4)
	80–84	200,019 (24.7)
	85–89	203,802 (25.2)
	90–94	125,209 (15.5)
	≥ 95	48,674 (6.0)
Time from admission to death (days)		196 (42–762)
Cumulative LOS (days)		79 (22–299)
	0–29	243,559 (30.1)
	30–59	114,228 (14.1)
	60–89	68,227 (8.4)
	90–179	110,183 (13.6)
	180–359	94,250 (11.6)
	360–999	112,981 (14.0)
	≥ 1,000	66,227 (8.2)
Cumulative medical cost (10^6^ KRW)		7.319 (2.033–27.621)
	0–5	341,513 (42.2)
	6–10	114,829 (14.2)
	11–19	106,640 (13.2)
	≥ 20	246,723 (30.5)
Death with LTCH stay	Yes	554,502 (68.5)
	No	255,203 (31.5)

Abbreviations: IQR, interquartile range; KRW, Korean Won; LOS, length of stay; LTCH, long-term care hospital.

Values are presented as numbers (%) or median (IQR).

Source: National Health Insurance Service (NHIS) claims data, 2014–2023.

### Transition from chronic morbidity to acute deterioration

**Fig 2:**
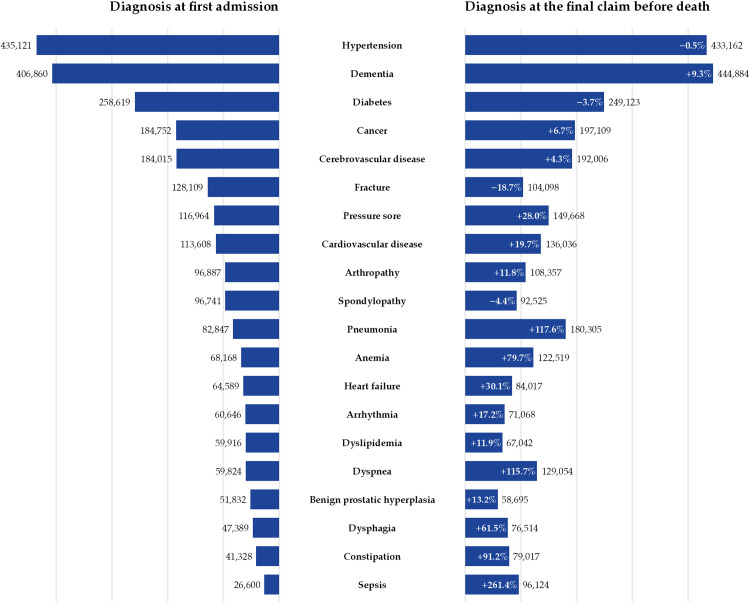
Most frequent diagnoses at the time of admission and at the final claim before death among older decedents in LTCHs (2015 – 2023, N = 809,705). The graph compares the prevalence of the top diagnoses recorded at first admission (left) with that at the final claim before death (right). Percentages indicate the relative rate of change between the two time points. Source: National Health Insurance Service (NHIS) claims data, 2014–2023.

Apart from general background comorbidities such as hypertension (53.7%) and diabetes (31.9%), the clinical profile at LTCH entry was characterized by major geriatric syndromes such as dementia (50.3%), cancer (22.8%), cerebrovascular disease (22.7%), and fractures (15.8%). By the final claim before death, a distinct shift toward acute and systemic deterioration occurred. While the burden of dementia remained high (55.0%), there was a surge in acute infectious conditions. Sepsis increased from 3.3% at admission to 11.9% at the final claim, while pneumonia increased from 10.2% to 22.3%. Furthermore, symptoms associated with end-of-life functional decline became prominent, such as dyspnea, constipation, anemia, and dysphagia. These diagnostic shifts are illustrated in Fig 2, and detailed numerical comparisons are provided in S2 Table.

## Discussion

This nationwide study (2014–2023) demonstrates the evolving role of Korean LTCHs as end-of-life care settings for older adults. While overall inpatient utilization grew gradually, a disproportionate rise in LTCH-associated deaths indicates an expanding role of these institutions in the final phase of life. Newly admitted patients, predominantly females in their early 80s, exhibited high sociodemographic vulnerability, marked by a disproportionate prevalence of Medical Aid beneficiaries and registered disabilities. Post-admission utilization was highly heterogeneous; despite a median survival of 196 days (IQR 42–762), cumulative LOS ranged from brief stays to prolonged institutionalization exceeding one year. Ultimately, 68.5% of decedents received care at an LTCH as their final place of care. Regarding the clinical trajectory, admissions were primarily driven by chronic geriatric syndrome, while the terminal phase was dominated by acute infectious and systemic conditions, notably sepsis, pneumonia, and dyspnea.

Temporal trends revealed a transient downturn in inpatient volumes during 2019–2020, likely attributable to the COVID-19 pandemic [21,22] and the 2019 payment reform [23]. However, interpreting these trends within the context of national demographics elucidates the evolving societal function of LTCHs. Notably, the substantially lower CAGR for new admissions compared with total inpatient volume suggests that the expansion of LTCH utilization may have been driven by prolonged institutional stays. This reflects the growing role of LTCHs as end-of-life care settings for older adults with chronic and complex medical needs. According to Statistics Korea, the proportion of older adults in the general population increased from 12.4% (6.5 million) in 2014 to 18.2% (9.7 million) in 2023 [24], which largely explains the rising proportion of older adults among LTCH admissions. However, the possibility that changes in referral patterns or healthcare utilization practices also contributed to these temporal trends cannot be ruled out. While the proportion of older adults admitted to LTCHs remained relatively stable at approximately 4% of the total older population throughout the decade, the sector’s contribution to end-of-life care has intensified disproportionately. During the same period, the annual number of deaths among older adults nationwide rose from 195,929 to 286,149 [24]. Among all older decedents in Korea, the proportion who utilized LTCH services during their year of death increased from 34.9% in 2014 to 39.2% in 2023. This divergence suggests that LTCHs have become an increasingly important stage in the end-of-life trajectory for older adults in Korea. Unlike nursing homes in Korea, LTCHs are physician-staffed medical institutions rather than welfare-oriented residential facilities, which may partly explain the increasing concentration of end-of-life care. Older adults with complex medical needs due to chronic illnesses may be more likely to remain in medically supervised settings near the end of life.

The sociodemographic profile of new admissions reveals that LTCHs serve a population situated at the intersection of social and physical vulnerability. The preponderance of women exceeds their proportion in the general older population (approximately 56.0% in 2023 [24]), which may partly reflect sex differences in longevity and disability burden among older adults, as women generally live longer but experience higher disability burdens [25,26]. The group also exhibited a marked concentration of registered disabilities (28.0%), compared with the national average for older adults (approximately 14.6% in 2023 [24]). The proportion of Medical Aid beneficiaries in our group (13.7%) was more than four times the rate in the general Korean population (approximately 3% [27]). Collectively, these findings indicate that socioeconomically and physically vulnerable older adults were disproportionately represented among LTCH admissions.

The trajectories of the decedent group illustrate that LTCHs have come to play an important role in the final phase of life, serving a dual function. While the patients spent a median of 79 days as an LTCH inpatient, the population was heterogeneous, comprising both short-stay users and patients with prolonged institutional stays. This bimodal pattern may reflect a duality in which shorter stays plausibly represent admissions for terminal care and longer stays reflect chronic institutional residence. However, the present descriptive analysis could not determine which patient characteristics distinguished short-stay from long-stay users, and these interpretations require further investigation. The wide variation in cumulative medical costs further indicates substantial heterogeneity in resource use. Future studies should examine the patient- and facility-level factors associated with differences in LOS and cumulative medical costs. Although admissions were largely driven by chronic multimorbidity, the period preceding death was characterized by a shift toward acute infectious and respiratory conditions. Such a shift is consistent with prior studies of the end-of-life course in older adults, in which terminal decline is frequently accompanied by acute infections such as pneumonia and sepsis [28–30]. This pattern suggests that for many older adults, the LTCH is no longer just a rehabilitation facility but a care model that integrates acute symptom management with palliative principles.

This study has several strengths. First, it used nationwide claims data covering the entire older population of Korea over a decade, thereby minimizing selection bias. Second, we tracked individuals from admission to death, providing the end-of-life trajectory of older patients in LTCHs. This offers a more complete picture of the role LTCHs play in the final phase of life. Taken together, these features provide an integrated, nationwide characterization of LTCH utilization that links trends in admissions and mortality with cumulative LOS, medical costs, and diagnostic change from admission to the final claim before death, which to our knowledge has not previously been reported.

There are several limitations. First, this study relies on administrative claims data, which inherently lack detailed clinical information such as functional status, cognitive impairment, and frailty, all crucial for understanding end-of-life trajectories. Consequently, symptom-based codes (e.g., dyspnea, dysphagia) may reflect billing practices rather than definitive clinical diagnoses, and diagnoses recorded on the final claim may not perfectly align with the actual cause or timing of death. Furthermore, because our diagnostic trajectory analysis was restricted to decedents, these clinical shifts may not be generalizable to patients who survived beyond the study period.

Second, this study was designed primarily as a descriptive population-level analysis. Without multivariable modeling, stratified analyses, or patient-level comparisons, we could not evaluate how clinical trajectories might differ across demographic subgroups (e.g., disability type and severity) or identify the characteristics that distinguished short-stay from long-stay users or accounted for the wide variation in cumulative costs. Furthermore, we did not account for facility-level variables (e.g., hospital size, ownership) or institutional clustering effects; thus, our aggregated estimates may mask substantial heterogeneity in care patterns and mortality across facilities. Additionally, given the massive sample size, even minor numerical differences may appear substantial, warranting cautious interpretation of their clinical significance.

Third, there are constraints related to operational definitions and data coverage. Because claims prior to 2014 were unavailable, some individuals classified as newly admitted may have had unobserved prior admissions, resulting in potential misclassification of newly admitted patients. Consequently, the time from initial LTCH admission to death may have been underestimated for these individuals, potentially limiting the accuracy of the reconstructed end-of-life trajectories. Furthermore, the interval between the final LTCH claim and death could not be quantified. Therefore, diagnoses recorded on the final claim may not accurately represent patients’ clinical conditions immediately before death, particularly among those who were discharged or received care elsewhere before death. This limitation introduces uncertainty into the timing and interpretation of the observed diagnostic shifts. Moreover, our operational definition of death within LTCHs may not perfectly capture the exact logistical circumstances surrounding discharge. Because the database lacks information on non-medical locations of death (e.g., homes or welfare-oriented nursing homes), we could not fully evaluate discharge destinations for patients who died outside LTCHs. Finally, our economic estimates likely underestimate the total financial burden because non-covered out-of-pocket expenses (e.g., caregiving, medical supplies) were unavailable.

## Conclusions

Our findings suggest that LTCHs have played an increasingly important role as end-of-life care settings in Korea, with more than two-thirds of decedents (68.5%) dying during LTCH stay. The findings also showed a shift from chronic geriatric conditions at admission toward more frequent acute infectious and systemic conditions in the final observed claims before death. Given the growing role of LTCHs in end-of-life care, policy reforms should strengthen palliative care integration, advance care planning, workforce training, and end-of-life care capacity within LTCHs to better address the complex needs of frail older adults.

## Supporting information

S1 Table Annual statistics for the total LTCH population (all ages), 2014–2023. (PDF)

S2 Table Detailed numerical comparison of recorded clinical conditions at admission and at the final claim before death among older decedents in LTCHs. (PDF)
