# Supplementary material for: Long-term care hospitals as end-of-life care settings in South Korea: A nationwide analysis of utilization patterns and clinical trajectories
Source: PLoS One. 2026 Jul 24;21(7):e0354271. doi: 10.1371/journal.pone.0354271 (PMC13399523; doi:10.1371/journal.pone.0354271)
Supplement: S1 Table — (PDF) [file pone.0354271.s001.pdf]

S1 Table. Annual statistics for the total LTCH population (all ages), 2014–2023.

Year	Total inpatients	New admissions	Deaths
2014	358,855	NA	76,694
2015	384,577	184,574	83,610
2016	409,857	185,721	87,513
2017	436,092	191,515	95,051
2018	463,669	201,464	102,137
2019	477,595	200,519	103,827
2020	444,372	167,104	103,015
2021	430,391	167,803	104,787
2022	464,520	200,716	130,999
2023	436,522	186,338	122,688

Abbreviations: LTCH, long-term care hospital; NA, not applicable.
New admissions data for 2014 are not available due to the washout period applied to define incident cases.
