# Supplementary material for: Long-term care hospitals as end-of-life care settings in South Korea: A nationwide analysis of utilization patterns and clinical trajectories
Source: PLoS One. 2026 Jul 24;21(7):e0354271. doi: 10.1371/journal.pone.0354271 (PMC13399523; doi:10.1371/journal.pone.0354271)
Supplement: S2 Table — (PDF) [file pone.0354271.s002.pdf]

S2 Table. Detailed numerical comparison of recorded clinical conditions at admission and at the final claim before death among older decedents in LTCHs.

Disease	ICD–10 codes	First claim N (%)	Final claim N (%)	Change (%)
Hypertension	I10–I13, I15	435,121 (53.7)	433,162 (53.5)	-0.5
Dementia	F00–F03	406,860 (50.2)	444,884 (54.9)	+9.3
Diabetes	E10–E14	258,619 (31.9)	249,123 (30.8)	-3.7
Cancer	C00–C26, C30–C34, C37–C41, C43–C58, C60–C71, C72–C86, C88, C90–C97, D00–D07, D09, D37–D48	184,752 (22.8)	197,109 (24.3)	+6.7
Cerebrovascular disease	I60–I69	184,015 (22.7)	192,006 (23.7)	+4.3
Fracture	S02, S12, S22, S32, S42, S52, S62, S72, S82, S92, T02, T08, T10, T12, T14.2	128,109 (15.8)	104,098 (12.9)	-18.7
Pressure sore	L89	116,964 (14.4)	149,668 (18.5)	+28.0
Cardiovascular disease	I20–I25, I42–I43, I50–I52	113,608 (14.0)	136,036 (16.8)	+19.7
Arthropathy	M00–M03, M05–M14, M15–M25	96,887 (12.0)	108,357 (13.4)	+11.8
Spondylopathy	M40–M43, M45–M51, M53–M54	96,741 (11.9)	92,525 (11.4)	-4.4
Pneumonia	J12–J18	82,847 (10.2)	180,305 (22.3)	+117.6
Anemia	D50–D53, D55–D77, D80–D84, D86, D89	68,168 (8.4)	122,519 (15.1)	+79.7
Heart failure	I50, I09.9, I11.0, I13.0, I13.2, I97.1	64,589 (8.0)	84,017 (10.4)	+30.1
Arrhythmia	I47–I49	60,646 (7.5)	71,068 (8.8)	+17.2
Dyslipidemia	E78	59,916 (7.4)	67,042 (8.3)	+11.9
Dyspnea	R06.0	59,824 (7.4)	129,054 (15.9)	+115.7
Benign prostatic hyperplasia	N40	51,832 (6.4)	58,695 (7.2)	+13.2
Dysphagia	R13	47,389 (5.9)	76,514 (9.4)	+61.5
Constipation	K59.09	41,328 (5.1)	79,017 (9.8)	+91.2
Sepsis	A02.1, A20.7, A22.7, A24.1, A26.7, A32.7, A40, A41, A42.7, A54.8, B00.7, B37.7, J95.0, O08.0, O75.3, O85, P36, R57.2, R65.0, R65.1, T80.2, T81.4, T88.0	26,600 (3.3)	96,124 (11.9)	+261.4

Abbreviations: ICD-10, International Statistical Classification of Diseases and Related Health Problems, 10th Revision; LTCH, long-term care hospital.
Change (%) represents the relative rate of change in prevalence between the two time points.
