# Supplementary material for: Long-term care hospitals as end-of-life care settings in South Korea: A nationwide analysis of utilization patterns and clinical trajectories
Source: PLoS One. 2026 Jul 24;21(7):e0354271. doi: 10.1371/journal.pone.0354271 (PMC13399523; doi:10.1371/journal.pone.0354271)
Supplement: Attachment — Submitted filename: PONE-D-26-12536.docx [file pone.0354271.s003.docx]

**Comments**

**Title**

The title is accurate. But "10-Year Utilization and End-of-Life Trajectories" – the "10-Year" part is redundant because they already give the period (2014-2023).

**Abstract**

- The authors mentioned "annual number of deaths involving LTCH use nearly doubled." Doubled from what? They don't give the baseline number. The CAGR is 5.66%, which is fine, but that doesn't obviously translate to "doubled" for a reader. How many years to double at that rate? just say.

- "Patients typically entered LTCHs in their early 80s", mean age at admission in the new admission cohort is 80.7, but in the decedent cohort it's 82.1. Which one is "typical"? They use the lower figure without explaining the discrepancy.

- The abstract was mentioned in the conclusion "policy to develop care models tailored to the evolving health status", that's so generic it could be written for almost any aging paper. No specific policy direction (staffing ratios? palliative training? payment reforms?).

**Introduction**

- The introduction correctly distinguishes LTCHs from nursing homes, one is physician, staffed, the other is welfare-focused. But then they never explore why that distinction might matter for end-of-life trajectories. If LTCHs have physicians and nursing homes don't, wouldn't you expect different death patterns? They mention this but don't do anything with it.

- They claim "previous studies have predominantly relied on limited cohorts" and cite references [9-15]. But reference [12] (Jeon et al. 2016) used national data. So that claim is overstated. They should acknowledge that national descriptive studies already exist, then specify what their study adds (e.g., the diagnostic shift analysis).

- Also, there's no clear research question or hypothesis. They list aims, but never say what they expected to find. For a descriptive study that's not strictly required, but it would strengthen the framing.

**Methods**

-Study design and data sources: The use of whole population NHIS claims data, 2014-2023 is a strength. But they don't report how many LTCH facilities were included or their distribution (size, ownership, geographic location). Facility-level variation could matter a lot for mortality and length of stay.

- Cohort definitions (annual inpatient cohort): They exclude individuals with missing or invalid data. How many? Not reported. If missing data patterns changed over time (e.g., more incomplete records during COVID), that could bias trend estimates.

- New admission cohort: They use a one-year washout period (2014). But someone admitted in late 2013 and re-admitted in early 2015 would be incorrectly counted as a new admission.

- Decedent cohort: They exclude individuals alive at the end of the study period (490,197, shown in Figure 1). That means the clinical trajectory analysis only applies to people who died. They then generalize these diagnostic shifts to "LTCH patients" as if it applies to survivors too. They need to state this limitation explicitly.

-Operational definitions of "death during LTCH stay": Defined as death date coinciding with the calculated discharge date (admission date + LOS – 1 day). That's an approximation. Administrative discharge dates don't always perfectly align with actual death dates (bodies held for family arrangements, etc.).

-Negative time values recoded as zero: They mention this but don't say what proportion of cases had negative values. Without that, readers can't judge how much this affects the accuracy of LOS and survival estimates.

-Diagnostic shifts: They use both primary and secondary ICD-10 codes from first admission and final claim before death. But the final claim might have been submitted days or even weeks before death if the patient was discharged elsewhere. They don't report the median time gap between final claim and death.

-HIRA classification guidelines, cited, but they don't explain which conditions were grouped into which categories. A reader unfamiliar with HIRA has no idea, for example, whether "sepsis" includes septic shock or just bacteremia.

-Statistical analysis: All descriptive. No hypothesis testing, no confidence intervals for differences (e.g., between admission and death diagnosis prevalence), no multivariable models. For a study with N > 800,000 decedents, the absence of even basic risk adjustment (e.g., stratified by age or sex) is a major shortcoming. They note this as a limitation, but it's presented as a choice rather than a flaw. They could have done stratified analyses.

-CAGR: Calculated, but no statistical test for trend (Joinpoint regression or Mann-Kendall). So the reader doesn't know if the observed increases are significant or just year-to-year noise.

-Exchange rate: 1 USD = 1,500 KRW. That's approximately the 2024-2025 rate. But costs span 2014-2023. Using a single exchange rate for a decade ignores inflation and currency fluctuations. This is methodologically incorrect. They should report costs in nominal KRW plus inflation-adjusted KRW (2023 basis) or use year-specific exchange rates.

**Results**

Table 1 (Annual trends):

- they don't report how many individuals were excluded because they had a prior admission in 2014. That number is essential to understand the size of the incident cohort.

-The CAGR for new admissions (0.87%) is much lower than for total inpatients (2.91%). That means growth is driven by longer stays, not more new patients. it suggests LTCHs are retaining patients longer, consistent with an end-of-life role. But they don't discuss it at all.

-The proportion of older adults among new admissions rose from 76.7% to 81.4%. They attribute this to aging demographics, but they never calculate the expected increase from demographic change alone. So we don't know if this increase is purely demographic or reflects a change in referral patterns.

Table 2 -

- Sociodemographic profile: Mean NHI premium reported as 85 ± 96 USD. The standard deviation exceeds the mean – that's a highly skewed distribution. Mean ± SD is inappropriate for skewed data. Median and IQR would be much better. The very low IQR lower bound (15 USD) suggests extreme poverty in a substantial subset .

-Disability status: 28.0% have registered disabilities. They compare this to the national average (14.6%) but don't provide the citation for that figure. Also, they don't specify type or severity of disability (physical vs. intellectual, severe vs. mild), which almost certainly affects LOS and mortality.

Table 3

-Mean cumulative LOS is 277.4 days. But 30.1% stayed less than 30 days. The mean is pulled upward by the long-stay tail (22.2% stayed ≥360 days). The median would be far lower – roughly 100 days, judging from the distribution. They use mean-dominated language in the text ("average 277 days"), which is misleading for such a skewed distribution.

-68.5% died during an LTCH stay. That means 31.5% died elsewhere after discharge. They never analyze where these people died (home, nursing home, acute hospital). Understanding discharge destination before death would tell us whether LTCHs are truly the final care setting or just one stop along the path.

- Mean cumulative medical cost was reported 16,600,IQR1,400-18, 400. Another skewed distribution–20.11,000 and 10.2% >$50,000. That's extreme variation. They don't comment on what this means for financial risk or equity.

- The percentage changes in Figure 2 (+261.4% for sepsis, +117.6% for pneumonia) look dramatic. But look at the absolute numbers: sepsis goes from 3.3% to 11.9%. That's an 8.6 percentage point increase. The percentage change is huge because the baseline is tiny. They should emphasize absolute changes, not just relative ones, to avoid overhyping.

-The figure has no error bars or confidence intervals. With N=809,705, even tiny differences are statistically significant, but clinical significance is another matter.

-Dyspnea, constipation, anemia, dysphagia – these are symptoms or signs, not diagnoses. In claims data, these may be billed as secondary diagnoses without specific etiologies. Treating them as diagnostic categories is conceptually problematic. Constipation at end of life is nearly universal, but its presence in claims depends on documentation practices, not clinical reality.

**Discussion**

-The first paragraph just repeats the results. A discussion should interpret, not restate.

-This key finding should have been in the results section: "the proportion of Korean older decedents who had utilized LTCH services in their year of death increased from 34.9% in 2014 to 39.2% in 2023."

-They invoke the "male-female health-survival paradox" to explain the female predominance. But 62.1% female vs. 56% in the general older population – the difference isn't that dramatic.

-They claim LTCHs serve as a "social safety net" because 13.7% are Medical Aid beneficiaries (vs. ~3% general population). But it is possible medical Aid beneficiaries may have fewer alternatives (private home care, family support) and enter LTCHs because of poverty, not because LTCHs are well-designed for them.

**Limitations:**

**-**They correctly note that final claim diagnoses reflect managed conditions, not underlying cause of death. But they don't mention that claims data lack functional status, cognitive impairment, or frailty measures, all critical for understanding end-of-life trajectories in LTCHs.

- they mention caregiving costs but ignore out-of-pocket costs for uncovered medications, supplies, or private duty nursing.

-The analysis is descriptive. That's not a limitation of the data. It's a limitation of their analytic choices.

**Conclusion**

-The evidence confirms that "LTCHs in Korea have become a primary setting for end-of-life care," but the conclusion does not provide the key number (68.5% die there).

-The policy recommendation ("develop care models tailored to the evolving health status") is too vague to be actionable.

-They did not measure patient-reported outcomes, family satisfaction, or any quality indicators of end-of-life care. Therefore, this claim "death with dignity" from this study is not based on evidence.

**References**

-Reference 4: DOI is incomplete .

-Reference 19: no URL or DOI. For a guideline document, provide a persistent identifier or repository link.

-Reference 26: Health Insurance Review & Assessment Service, 2025 – that's very recent (published after the study period ended). Was it available when they wrote the manuscript?

-Several references (9, 10, 11, 13, 14, 15) are older (>5 years) and focused on small cohorts.
