# Supplementary material for: Long-term care hospitals as end-of-life care settings in South Korea: A nationwide analysis of utilization patterns and clinical trajectories
Source: PLoS One. 2026 Jul 24;21(7):e0354271. doi: 10.1371/journal.pone.0354271 (PMC13399523; doi:10.1371/journal.pone.0354271)
Supplement: Attachment — Submitted filename: Response_to_reviewers_260601.docx [file pone.0354271.s005.docx]

**Point-by-point response**

**Journal Requirements**

**Comment 1.**

Please ensure that your manuscript meets PLOS ONE’s style requirements, including those for file naming.

**Response:**

We appreciate the journal’s guidance. The manuscript and submission files were carefully revised and formatted in strict accordance with the 'Manuscript body formatting guidelines' and the 'Title, author, affiliations formatting guidelines', including the specific file naming requirements.

**Comment 2.**

Please update your submission to use the PLOS LaTeX template.

**Response:**

We appreciate the journal’s guidance. We have fully updated our submission using the official PLOS LaTeX template. Additionally, we utilized the latexdiff tool to generate the Tracked Changes version of the manuscript.

**Comment 3.**

Please provide an amended statement that declares all funding or sources of support. Please also include the statement “There was no additional external funding received for this study.”

**Response:**

We appreciate the journal’s guidance regarding the Funding Statement. We added the sentence (“There was no additional external funding received for this study.”) in the Funding Statement.

**Revision:**

*Funding*

This work was supported by the Korea Health Technology R&D Project through the Korea Health Industry Development Institute (KHIDI, https://www.khidi.or.kr), funded by the Ministry of Health & Welfare, Republic of Korea (Grant No. RS-2024-00441486 awarded to SS). The funders had no role in the study design, data collection and analysis, decision to publish, or preparation of the manuscript.

↓

This work was supported by the Korea Health Technology R&D Project through the Korea Health Industry Development Institute (KHIDI, https://www.khidi.or.kr), funded by the Ministry of Health & Welfare, Republic of Korea (Grant No. RS-2024-00441486 awarded to SS). The funders had no role in the study design, data collection and analysis, decision to publish, or preparation of the manuscript. There was no additional external funding received for this study.

**Comment 4.**

We note that you have indicated that there are restrictions to data sharing for this study. For studies involving human research participant data or other sensitive data, we encourage authors to share de-identified or anonymized data. However, when data cannot be publicly shared for ethical reasons, we allow authors to make their data sets available upon request. For information on unacceptable data access restrictions, please see http://journals.plos.org/plosone/s/data-availability#loc-unacceptable-data-access-restrictions.

Before we proceed with your manuscript, please address the following prompts:

a) If there are ethical or legal restrictions on sharing a de-identified data set, please explain them in detail (e.g., data contain potentially identifying or sensitive patient information, data are owned by a third-party organization, etc.) and who has imposed them (e.g., a Research Ethics Committee or Institutional Review Board, etc.). Please also provide contact information for a data access committee, ethics committee, or other institutional body to which data requests may be sent.

b) If there are no restrictions, please upload the minimal anonymized data set necessary to replicate your study findings to a stable, public repository and provide us with the relevant URLs, DOIs, or accession numbers. Please see http://www.bmj.com/content/340/bmj.c181.long for guidelines on how to de-identify and prepare clinical data for publication. For a list of recommended repositories, please see https://journals.plos.org/plosone/s/recommended-repositories. You also have the option of uploading the data as Supporting Information files, but we would recommend depositing data directly to a data repository if possible.

Please update your Data Availability statement in the submission form accordingly.

**Response:**

We appreciate the journal’s guidance regarding the Data Availability Statement. We revised the Data Availability Statement to clarify the nature of the restrictions, identify the responsible institution, and provide information regarding the procedure for data access requests.

**Revision:**

*Data Availability*

The data used in this study are derived from national health insurance claims and are not publicly available due to legal and regulatory restrictions. Access to the data requires approval from the data provider and is limited to authorised researchers under strict confidentiality agreements.

↓

The data used in this study are derived from the National Health Insurance Service (NHIS) claims database of Korea and contain potentially sensitive patient information. Due to legal restrictions imposed by the NHIS, the data cannot be publicly shared. Access to the data is available only to qualified researchers upon reasonable request and approval from the NHIS Data Sharing Service. Researchers may apply for access through the NHIS website (https://nhiss.nhis.or.kr). All data were accessed in a de-identified form under strict confidentiality agreements.

**Comment 5.**

Please include captions for your Supporting Information files at the end of your manuscript, and update any in-text citations accordingly.

**Response:**

We appreciate the journal’s guidance. We added captions for all Supporting Information files at the end of the manuscript and updated the corresponding in-text citations in the Results section.

**Revision:**

*Supporting Information*

S1 Table. Annual statistics for the total LTCH population (all ages), 2014–2023.

S2 Table. Detailed numerical comparison of recorded clinical conditions at admission and near death among older decedents in LTCHs.

**Comment 6.**

If the reviewer comments include a recommendation to cite specific previously published works, please review and evaluate these publications to determine whether they are relevant and should be cited. There is no requirement to cite these works unless the editor has indicated otherwise.

**Response:**

We appreciate the journal’s guidance. We reviewed the reviewer’s comments and found no recommendations to cite specific previously published works.

**Reviewer 1**

**Comment 1.**

Congratulations. It is an excellent article. The methods section is complete and it can be replicated in another context. The discussion is complete and compares the findings. Limitations are highlighted. Some references have more than 10 years, but they are useful for the article. Only I suggest adding the source in tables and figures.

**Response:**

We appreciate the reviewer’s helpful suggestion. We added the data source information in the footnotes of all the tables and figures.

**Revision:**

Source: National Health Insurance Service (NHIS) claims data, 2014–2023.

**Reviewer 2**

**Comment 1.**

The title is accurate. But "10-Year Utilization and End-of-Life Trajectories" – the "10-Year" part is redundant because they already give the period (2014-2023).

**Response:**

We appreciate the reviewer’s helpful suggestion. We revised the title to improve conciseness and clarity.

**Revision:**

*Title*

10-Year Utilization and End-of-Life Trajectories in Long-Term Care Hospitals of South Korea

↓

Utilization and end-of-life trajectories in long-term care hospitals of South Korea

**Comment 2.**

The authors mentioned “annual number of deaths involving LTCH use nearly doubled.” Doubled from what? They don’t give the baseline number. The CAGR is 5.66%, which is fine, but that doesn’t obviously translate to “doubled” for a reader.

**Response:**

We appreciate the reviewer’s helpful comment. We revised the relevant sentences in the Abstract and Discussion as follows.

**Revision:**

*Abstract*

… the annual number of deaths involving LTCH use nearly doubled over the decade.

↓

… the annual number of deaths involving LTCH use increased from 68,357 in 2014 to 112,157 in 2023 over the decade

*Discussion (Deleted)*

… the annual number of deaths involving LTCH use nearly doubled over the decade.

**Comment 3.**

“Patients typically entered LTCHs in their early 80s”, mean age at admission in the new admission cohort is 80.7, but in the decedent cohort it’s 82.1. Which one is “typical”? They use the lower figure without explaining the discrepancy.

**Response:**

We appreciate the reviewer’s careful observation. To improve clarity, we revised the sentence as follows.

**Revision:**

*Abstract*

Patients typically entered LTCHs in their early 80s …

↓

Patients were newly admitted to LTCHs in their early 80s (median age 81 years, IQR 75–86) …

*Discussion*

New admissions typically occurred in the early 80s …

↓

Newly admitted patients, predominantly females in their early 80s, exhibited …

**Comment 4.**

The abstract conclusion mentioned “policy to develop care models tailored to the evolving health status”, that’s so generic it could be written for almost any aging paper. No specific policy direction (staffing ratios? palliative training? payment reforms?).

**Response:**

We appreciate the reviewer’s constructive comment. We revised the Abstract and Conclusion to provide more concrete policy implications as follows.

**Revision:**

*Abstract*

… a policy to develop care models tailored to the evolving health status of older adults is needed.

↓

… policies supporting palliative care integration and end-of-life care capacity within LTCHs are needed.

*Conclusion*

…. policy reforms must develop care models tailored to the evolving health status of older adults.

↓

…. policy reforms should strengthen palliative care integration, advance care planning, workforce training, and end-of-life care capacity within LTCHs to better address the complex needs of frail older adults.

**Comment 5.**

The introduction correctly distinguishes LTCHs from nursing homes, one is physician-staffed, the other is welfare-focused. But then they never explore why that distinction might matter for end-of-life trajectories. If LTCHs have physicians and nursing homes don’t, wouldn’t you expect different death patterns? They mention this but don’t do anything with it.

**Response:**

We appreciate the reviewer’s insightful comment. We added the following sentences in the Discussion.

**Revision:**

*Discussion (Newly added)*

Unlike nursing homes in Korea, LTCHs are physician-staffed medical institutions rather than welfare-oriented residential facilities, which may partly explain the increasing concentration of end-of-life care. Older adults with complex medical needs due to chronic illnesses may be more likely to remain in medically supervised settings near the end of life.

**Comment 6.**

They claim “previous studies have predominantly relied on limited cohorts” and cite references [9–15]. But reference [12] (Jeon et al. 2016) used national data. So that claim is overstated. They should acknowledge that national descriptive studies already exist, then specify what their study adds (e.g., the diagnostic shift analysis).

**Response:**

We appreciate the reviewer’s helpful comment. We revised the sentence to properly acknowledge prior work while explicitly highlighting what our study adds.

**Revision:**

*Introduction*

Although previous studies on Korean LTCHs have investigated specific clinical outcomes (e.g., length of stay (LOS) or functional status), they have predominantly relied on limited cohorts [9–15].

↓

Although previous studies on Korean LTCHs have investigated specific clinical outcomes (e.g., length of stay [LOS] or functional status), they often lacked a comprehensive evaluation of longitudinal end-of-life trajectories and diagnostic shifts [9–15].

**Comment 7.**

Also, there’s no clear research question or hypothesis. They list aims, but never say what they expected to find. For a descriptive study that’s not strictly required, but it would strengthen the framing.

**Response:**

We appreciate the reviewer’s helpful suggestion. We added a sentence at the end of the Introduction to clarify our expectations regarding the expanding role of LTCHs and the specific clinical trajectories of older adults.

**Revision:**

*Introduction (Newly added)*
We anticipated that LTCHs in Korea would increasingly serve as primary end-of-life care settings, managing high mortality burdens and distinct clinical trajectories beyond their traditional role in subacute rehabilitation.

**Comment 8.**

Study design and data sources: The use of whole population NHIS claims data, 2014–2023 is a strength. But they don’t report how many LTCH facilities were included or their distribution (size, ownership, geographic location). Facility-level variation could matter a lot for mortality and length of stay.

**Response:**

We deeply appreciate the reviewer’s insightful comment and completely agree that facility-level characteristics are important factors that likely drive substantial variation in mortality and length of stay. However, incorporating facility-level variables would require a complex multi-level or hierarchical modeling framework, which would fundamentally shift the scope of this paper. Therefore, we revised the limitations section to clarify this concern.

**Revision:**

*Discussion (Newly added)*

Furthermore, we did not account for facility-level variables (e.g., hospital size, ownership) or institutional clustering effects; thus, our aggregated estimates may mask substantial heterogeneity in care patterns and mortality across facilities.

**Comment 9.**

Cohort definitions (annual inpatient cohort): They exclude individuals with missing or invalid data. How many? Not reported. If missing data patterns changed over time (e.g., more incomplete records during COVID), that could bias trend estimates.

**Response:**

We appreciate the reviewer’s important comment. There were zero cases excluded due to missing or invalid data for the study's key variables, even though we originally planned it. As indicated in the Results section, 312,618 individuals were excluded from the annual inpatient group to form the newly admitted inpatient group because they had prior admission records during the 2014 washout period. To prevent confusion, we have removed the relevant sentence in the Statistical Analysis sections and revised the relevant phrases in Figure 1.

**Revision:**

*Materials and methods (Deleted)*

We performed a complete-case analysis, excluding individuals with missing or invalid data on key variables.

*Figure 1*

Excluded: Patients with previous records or invalid data (N = 312,618)

↓

Excluded: Patients with prior records during the washout period (N = 312,618)

**Comment 10.**

New admission cohort: They use a one-year washout period (2014). But someone admitted in late 2013 and re-admitted in early 2015 would be incorrectly counted as a new admission.

**Response:**

We appreciate the reviewer’s important observation. Because claims data prior to 2014 were unavailable, some individuals classified as newly admitted patients may have had LTCH admissions before the washout period. Therefore, we clarified the description of the washout approach in the Methods section and additionally acknowledged this issue as a study limitation.

**Revision:**

*Methods*

(ensuring a minimum one-year washout period)

↓

(using a minimum one-year washout period based on available claims data)

*Discussion (Newly added)*

Because claims prior to 2014 were unavailable, some individuals classified as newly admitted may have had unobserved prior admissions.

**Comment 11.**

Decedent cohort: They exclude individuals alive at the end of the study period (490,197, shown in Figure 1). That means the clinical trajectory analysis only applies to people who died. They then generalize these diagnostic shifts to “LTCH patients” as if it applies to survivors too. They need to state this limitation explicitly

**Response:**

We appreciate the reviewer’s important comment. We acknowledged this issue in the Limitations section.

**Revision:**

*Discussion (Newly added)*

Furthermore, because our diagnostic trajectory analysis was restricted to decedents, these clinical shifts may not be generalizable to patients who survived beyond the study period.

**Comment 12.**

Operational definitions of “death during LTCH stay”: Defined as death date coinciding with the calculated discharge date (admission date + LOS – 1 day). That’s an approximation. Administrative discharge dates don’t always perfectly align with actual death dates (bodies held for family arrangements, etc.).

**Response:**

We appreciate the reviewer’s important comment. We additionally acknowledged it as a limitation of the study.

**Revision:**

*Discussion (Newly added)*

Moreover, our operational definition of death within LTCHs may not perfectly capture the exact logistical circumstances surrounding discharge.

**Comment 13.**

Negative time values recoded as zero: They mention this but don’t say what proportion of cases had negative values. Without that, readers can’t judge how much this affects the accuracy of LOS and survival estimates.

**Response:**

We appreciate the reviewer’s important comment. We additionally quantified the number of cases with negative time values in the calculation of time from admission to death, which were only 8 cases (<0.001% of the decedent group). Therefore, we believe that the impact on LOS and survival estimates was negligible. We newly added this to the Results section.

**Revision:**

*Results (Newly added)*

The number of cases with negative time values in the calculation of time from admission to death was only 8 cases in the decedent group.

**Comment 14.**

Diagnostic shifts: They use both primary and secondary ICD-10 codes from first admission and final claim before death. But the final claim might have been submitted days or even weeks before death if the patient was discharged elsewhere. They don’t report the median time gap between final claim and death.

**Response:**

We sincerely appreciate the reviewer’s insightful comment. While we successfully calculated the cumulative time from the initial admission to death, we unfortunately cannot quantify the median time gap between the final claim and the date of death. This is because our authorized access period to the NHIS secure data platform for this project has now expired. Therefore, we acknowledged this as a limitation.

**Revision:**

*Discussion*

First, diagnoses recorded on the final claim were used as a proxy for the clinical condition near death. These reflect the burden of disease managed by the LTCH at the end of life rather than the medically certified underlying cause of death.

↓

…. and diagnoses recorded on the final claim may not perfectly align with the actual cause or timing of death.

**Comment 15.**

HIRA classification guidelines are cited, but the manuscript does not explain which conditions were grouped into which categories. A reader unfamiliar with the HIRA system may not understand how categories such as “sepsis” were operationally defined.

**Response:**

We appreciate the reviewer’s helpful comment. The HIRA disease classification (based on ICD-10 codes) is a standardized national guideline that reflects the consensus of HIRA expert committees, working-level staff, and relevant specialized medical societies. It is widely used as the standard for calculating healthcare statistics in Korea. Although including these guidelines in the manuscript or appendices would be overly extensive, the full document is publicly accessible online, ensuring complete methodological reproducibility. Furthermore, we would like to note that the exact ICD-10 codes used to define each diagnostic category are explicitly detailed in S2 Table.

**Comment 16.**

Statistical analysis: All analyses were descriptive, with no hypothesis testing, confidence intervals, or multivariable modelling. Given the large nationwide dataset, the absence of even basic stratified analyses (e.g., by age or sex) is a major shortcoming. Although this was mentioned as a limitation, it was presented more as a methodological choice than as a limitation.

**Response:**

We appreciate the reviewer’s thoughtful comment. We revised the Limitations section to more explicitly acknowledge this methodological limitation.

**Revision:**

*Discussion*

Finally, our analysis was descriptive by design. We did not employ multivariable modeling to identify risk factors associated with specific outcomes.

↓

Second, this study was designed primarily as a descriptive population-level analysis. Without multivariable modeling or stratified analyses, we could not evaluate how clinical trajectories might differ across specific demographic subgroups, …

**Comment 17.**

CAGR: Calculated, but no statistical test for trend (Joinpoint regression or Mann-Kendall). So the reader doesn’t know if the observed increases are significant or just year-to-year noise.

**Response:**

We appreciate the reviewer’s important comment regarding the assessment of temporal trends. We newly added regression analyses across the entire study period using all annual data points.

**Revision:**

*Materials and methods*

We summarized annual counts and proportions by calendar year and calculated compound annual growth rates (CAGR) to quantify trends over the study period.

↓

We summarized annual counts and proportions by calendar year and calculated compound annual growth rates (CAGR) based on the first and last observed annual values to quantify overall temporal changes during the study period. In addition, linear regression analyses were performed across the entire study period to assess temporal trends.

*Table 1*

| Year | Total inpatients | New admissions | Deaths |
| --- | --- | --- | --- |
| … | … | … | … |
| CAGR (%) | 2.91 | 0.87 | 5.66 |
| p for trend | 0.006 | 0.727 | < 0.001 |

… CAGR was calculated using the first and last observed annual values. P for trend was obtained using linear regression analyses across annual data points. …

**Comment 18.**

Exchange rate: 1 USD = 1,500 KRW. That’s approximately the 2024–2025 rate. However, costs span 2014–2023. Using a single exchange rate across a decade ignores inflation and currency fluctuations. They should report costs in nominal KRW plus inflation-adjusted KRW (2023 basis) or use year-specific exchange rates.

**Response:**

We appreciate the reviewer’s important methodological comment. To address this concern, we have removed the fixed USD conversion entirely. Instead, we now report all medical costs and insurance premiums in their original nominal Korean Won (KRW).

**Revision:**

*Materials and methods (Deleted)*

Medical costs were converted to US dollars (USD) by applying an exchange rate of 1 USD = 1,500 KRW.

*Results*

Regarding expenditures, the mean cumulative medical cost from admission to death was approximately $16,600, and 10.2% of decedents incurred expenses exceeding $50,000.

↓

Regarding expenditures, the median cumulative medical cost from admission to death was approximately 7.3 million Korean Won.

*Table 2*

| **Characteristic** | | **N (%) or Mean (IQR)** |
| --- | --- | --- |
| … |  |  |
| Premium (USD) |  | 85 ± 96 (15 – 127) |
| … |  |  |

↓

| **Characteristic** | | **N (%) or Median (IQR)** |
| --- | --- | --- |
| … |  |  |
| Premium (KRW) |  | 97,490 (23,240 – 189,890) |
| … |  |  |

*Table 3*

| **Characteristic** | | **N (%) or Mean ± SD (IQR)** |
| --- | --- | --- |
| … |  |  |
| Cumulative medical cost (USD) |  | 16,600 ± 27,400 (1,400 – 18,400) |
|  | 0 – 999 | 162,874 (20.1) |
|  | 1,000 – 4,999 | 245,952 (30.4) |
|  | 5,000 – 9,999 | 111,467 (13.8) |
|  | 10,000 – 19,999 | 97,878 (12.1) |
|  | 20,000 – 49,999 | 108,612 (13.4) |
|  | ≥ 50,000 | 82,922 (10.2) |
| … |  | … |

…. Values are presented as numbers (%) for categorical variables and mean ± standard deviation (interquartile range) for continuous variables….

↓

| **Characteristic** | | **N (%) or Median (IQR)** |
| --- | --- | --- |
| … |  |  |
| Cumulative medical cost (10^6^ KRW) |  | 7.319 (2.033 – 27.621) |
|  | 0 – 5 | 341,513 (42.2) |
|  | 6 – 10 | 114,829 (14.2) |
|  | 11 – 19 | 106,640 (13.2) |
|  | ≥ 20 | 246,723 (30.5) |
| … |  | … |

**Comment 19.**

They do not report how many individuals were excluded because they had a prior admission in 2014. That number is essential to understand the size of the incident cohort.

**Response:**

We appreciate the reviewer’s careful comment. Although the exact number of excluded individuals (N = 312,618) was originally presented in Figure 1, we have now included this number in the Results section.

**Revision:**

*Results*

After applying a washout period, we identified 1,299,902 individuals for the newly admitted inpatient group (2015–2023).

↓

After excluding 312,618 patients with prior admissions during the washout period, we identified 1,299,902 individuals in the newly admitted inpatient group (2015–2023).

**Comment 20.**

The CAGR for new admissions (0.87%) is much lower than for total inpatients (2.91%). This suggests that growth in LTCH utilization may be driven more by prolonged stays than by increasing numbers of new patients, consistent with an expanding end-of-life care role. However, this point is not discussed in the manuscript.

**Response:**

We appreciate the reviewer’s insightful comments. We added this to the Discussion section.

**Revision:**

*Discussion (Newly added)*
Notably, the substantially lower CAGR for new admissions compared with total inpatient volume suggests that the expansion of LTCH utilization may have been driven by prolonged institutional stays. This reflects the growing role of LTCHs as end-of-life care settings for older adults with chronic and complex medical needs.

**Comment 21.**

The proportion of older adults among new admissions rose from 76.7% to 81.4%. The manuscript attributes this increase to aging demographics, but it does not evaluate whether the observed increase can be explained by demographic aging alone versus possible changes in referral patterns.

**Response:**

We appreciate the reviewer’s thoughtful comment. We have revised the Discussion to acknowledge potential changes in referral patterns and healthcare utilization.

**Revision:**

*Discussion*

According to Statistics Korea, the whole older population expanded from 6.5 million in 2014 to 9.7 million in 2023 [23].

↓

According to Statistics Korea, the proportion of older adults in the general population increased from 12.4% (6.5 million) in 2014 to 18.2% (9.7 million) in 2023 [23], which largely explains the rising proportion of older adults among LTCH admissions. However, the possibility that changes in referral patterns or healthcare utilization practices also contributed to these temporal trends cannot be ruled out.

**Comment 22.**

Mean NHI premium was reported as 85 ± 96 USD. Because the standard deviation exceeds the mean, the distribution appears highly skewed. Mean ± SD may therefore be inappropriate, and median with IQR would better represent the distribution. The very low IQR lower bound also suggests substantial socioeconomic vulnerability in a subset of patients.

**Response:**

We appreciate the reviewer’s insightful comments. We agree that several variables, including NHI premium, showed skewed distributions. Therefore, we revised Tables 2 and 3 to present continuous variables as median (IQR) instead of mean ± standard deviation.

**Revision:**

*Table 2*

| **Characteristic** | | **N (%) or Mean (IQR)** |
| --- | --- | --- |
| … |  |  |
| Age at admission (years) |  | 80.7 ± 7.7 (75 – 86) |
| … |  |  |
| Premium (USD) |  | 85 ± 96 (15 – 127) |
| … |  |  |

… Values are presented as numbers (%) or means ± SD (IQR). …

↓

| **Characteristic** | | **N (%) or Median (IQR)** |
| --- | --- | --- |
| … |  |  |
| Age at admission (years) |  | 81 (75 – 86) |
| … |  |  |
| Premium (KRW) |  | 97,490 (23,240 – 189,890) |
| … |  |  |

… Values are presented as numbers (%) or median (IQR). …

*Table 3*

| **Characteristic** | | **N (%) or Mean ± SD (IQR)** |
| --- | --- | --- |
| Age at admission (years) |  | 82.1 ± 7.4 (77 – 87) |
|  |  | … |
| Age at death (years) |  | 83.4 ± 7.5 (79 – 89) |
|  |  | … |
| Time from admission to death (days) | | 506.6 ± 650.5 (42 – 762) |
| Cumulative LOS (days) |  | 277.4 ± 458.2 (22 – 299) |
|  |  | … |
| Cumulative medical cost (USD) |  | 16,600 ± 27,400 (1,400 – 18,400) |
|  |  | … |

…. Values are presented as numbers (%) for categorical variables and mean ± standard deviation (interquartile range) for continuous variables….

↓

| **Characteristic** | | **N (%) or Median (IQR)** |
| --- | --- | --- |
| Age at admission (years) |  | 82 (77 – 87) |
|  |  | … |
| Age at death (years) |  | 84 (79 – 89) |
|  |  | … |
| Time from admission to death (days) | | 196 (42 – 762) |
| Cumulative LOS (days) |  | 79 (22 – 299) |
|  |  | … |
| Cumulative medical cost (10^6^ KRW) |  | 7.319 (2.033 – 27.621) |
|  |  | … |

… Values are presented as numbers (%) or median (IQR) ….

**Comment 23.**

Disability prevalence was compared with the national average (14.6%), but the citation for that figure was not clearly presented. In addition, the manuscript does not specify disability type or severity, which may influence length of stay and mortality.

**Response:**

We sincerely appreciate the reviewer’s careful review. Regarding the national disability prevalence estimate (14.6%), we respectfully clarify that the source was cited as Reference 23 (Statistics Korea. Korean Statistical Information Service (KOSIS). <https://kosis.kr/eng/>). However, we admit that the specific type and severity of disability likely influence the length of stay and mortality. To address this concern, we have acknowledged tthis in the Discussion section.

**Revision:**

*Discussion (Newly added)*
… we could not evaluate how clinical trajectories might differ across specific demographic subgroups, including detailed characteristics such as disability type and severity.

**Comment 24.**

Mean cumulative length of stay (LOS) was reported as 277.4 days, despite the highly skewed distribution in which 30.1% of patients stayed less than 30 days while 22.2% stayed ≥360 days. Mean-based descriptions may therefore be misleading, and median values would better represent the central tendency.

**Response:**

We appreciate the reviewer’s insightful comments. We revised Tables 2 and 3 to report medians and interquartile ranges (IQRs) instead of means ± standard deviations. We also revised the Results and Discussion sections to avoid mean-dominated wording and to better reflect the heterogeneous distribution of LTCH utilization.

**Revision:**

*Results*

Within this timeframe, patients spent an average of 277.4 days (approximately 9 months) as LTCH inpatients.

↓

Within this timeframe, the median cumulative LOS was 79 days (IQR 22–299), although utilization patterns were highly variable.

*Discussion*

… they spent more than half of this remaining time (approximately 9 months) hospitalized in LTCHs

↓

Post-admission utilization was highly heterogeneous; despite a median survival of 196 days (IQR 42–762), cumulative LOS ranged from brief stays to prolonged institutionalization exceeding one year.

**Comment 25.**

Although 68.5% of patients died during an LTCH stay, 31.5% died elsewhere after discharge. The manuscript does not evaluate where these patients died (e.g., home, nursing home, acute hospital). Understanding discharge destination would help clarify whether LTCHs function as the final place of care or as one stage in the end-of-life trajectory.

**Response:**

We appreciate the reviewer’s important comment. We were unable to evaluate post-discharge settings because the administrative claims database used in this study does not contain information on discharge destinations or on specific locations of death outside medical institutions.

**Revision:**

*Discussion (Newly added)*
Because the database lacks information on non-medical locations of death (e.g., homes or welfare-oriented nursing homes), we could not fully evaluate discharge destinations for patients who died outside LTCHs.

**Comment 26.**

Cumulative medical costs showed substantial variation, with a wide interquartile range and both very low-cost and very high-cost subgroups. However, the manuscript does not discuss the implications of this heterogeneity for financial burden or equity.

**Response:**

We appreciate the reviewer’s insightful comment. We added a corresponding interpretation to the Discussion section to acknowledge the potential implications of this variability.

**Revision:**

*Discussion (Newly added)*
The wide variation in cumulative medical costs further suggests substantial heterogeneity in end-of-life care utilization and may reflect differences in clinical complexity, care needs, and financial burden among LTCH patients.

**Comment 27.**

The percentage increases shown in Figure 2 (e.g., +261.4% for sepsis and +117.6% for pneumonia) appear dramatic because the baseline prevalence at admission was relatively low. Relative percentage changes alone may overemphasize these trends, and absolute changes should also be considered.

**Response:**

We appreciate the reviewer’s insightful comment. We revised the Results section as follows.

**Revision:**

*Results*

Sepsis showed the largest relative increase (+261.4%, rising from 3.3% to 11.9%), followed by pneumonia (+117.6%, from 10.2% to 22.3%). Furthermore, symptoms associated with end-of-life functional decline became prominent. Dyspnea (+115.7%), constipation (+91.2%), anemia (+79.7%), and dysphagia (+61.5%) increased sharply relative to admission.

↓

Sepsis increased from 3.3% at admission to 11.9% near death, while pneumonia increased from 10.2% to 22.3%. Furthermore, symptoms associated with end-of-life functional decline became prominent, such as dyspnea, constipation, anemia, and dysphagia.

**Comment 28.**

The figure does not present error bars or confidence intervals. Given the very large sample size (N = 809,705), even small differences may be statistically significant, whereas the clinical significance of these differences may be limited.

**Response:**

We appreciate the reviewer’s thoughtful comment. We added a clarifying statement to the Limitations section.

**Revision:**

*Discussion (Newly added)*
Additionally, given the massive sample size, even minor numerical differences may appear substantial, warranting cautious interpretation of their clinical significance.

**Comment 29.**

Conditions such as dyspnea, constipation, anemia, and dysphagia are symptoms or clinical signs rather than definitive diagnoses. In claims data, these codes may reflect documentation or billing practices rather than underlying disease entities, making their interpretation as diagnostic categories conceptually challenging.

**Response:**

We appreciate the reviewer’s insightful comment. To clarify this point, we added a statement to the Limitations section.

**Revision:**

*Discussion (Newly added)*
Consequently, symptom-based codes (e.g., dyspnea, dysphagia) may reflect billing practices rather than definitive clinical diagnoses, …

**Comment 30.**

The first paragraph of the Discussion primarily restates the results rather than interpreting their implications.

**Response:**

We appreciate the reviewer’s helpful comment. We revised the paragraph to place greater emphasis on the broader interpretation and implications of the findings, particularly regarding the evolving role of LTCHs in end-of-life care for older adults.

**Revision:**

This nationwide study of older adults in Korean LTCHs (2014–2023) provides comprehensive evidence of the institution’s evolving role in geriatric and end-of-life care. First, the scale of mortality involving LTCHs has expanded dramatically. While the volume of older inpatients grew steadily, the annual number of deaths involving LTCH use nearly doubled over the decade. Second, the population entered with high sociodemographic vulnerability. New admissions typically occurred in the early 80s and were predominantly female, with a disproportionately high prevalence of Medical Aid beneficiaries and registered disabilities. Third, patients exhibited an intensive end-of-life utilization pattern. On average, individuals survived for approximately 17 months following their initial admission, and they spent more than half of this remaining time (approximately 9 months) hospitalized in LTCHs. Ultimately, more than two-thirds of the cohort died via LTCHs, marking it as their final place of care. Finally, a distinct clinical shift occurred from admission to death. While admissions were largely driven by major geriatric syndromes (e.g., dementia, cancer, cerebrovascular disease, fracture), the period near death was dominated by a surge in acute infectious and systemic conditions, particularly sepsis, pneumonia, and dyspnea.

↓

This nationwide study (2014–2023) demonstrates the evolving role of Korean LTCHs in primary end-of-life care settings for older adults. While overall inpatient utilization grew gradually, a disproportionate rise in LTCH-associated deaths indicates an expanding role of these institutions in the final phase of life. Newly admitted patients, predominantly females in their early 80s, exhibited high sociodemographic vulnerability, marked by a disproportionate prevalence of Medical Aid beneficiaries and registered disabilities. Post-admission utilization was highly heterogeneous; despite a median survival of 196 days (IQR 42–762), cumulative LOS ranged from brief stays to prolonged institutionalization exceeding one year. Ultimately, 68.5% of decedents received care at an LTCH as their final place of care. Regarding the clinical trajectory, admissions were primarily driven by chronic geriatric syndrome, while the terminal phase was dominated by acute infectious and systemic conditions, notably sepsis, pneumonia, and dyspnea.

**Comment 31.**

The finding that “the proportion of Korean older decedents who had utilized LTCH services in their year of death increased from 34.9% in 2014 to 39.2% in 2023” is an important result and should be presented in the Results section rather than only in the Discussion.

**Response:**

We appreciate the reviewer’s helpful comment. We added the corresponding statement to the Discussion section.

**Revision:**

*Discussion* (Newly added)
Among all older decedents in Korea, the proportion who utilized LTCH services during their year of death increased from 34.9% in 2014 to 39.2% in 2023.

**Comment 32.**

The manuscript invokes the “male–female health–survival paradox” to explain the female predominance in LTCH admissions. However, the difference between the study population (62.1% female) and the general older population (approximately 56% female) may not be substantial enough to justify such a strong theoretical interpretation.

**Response:**

We appreciate the reviewer’s thoughtful comment. We revised the Discussion section to adopt a more moderate interpretation, focusing on sex differences in longevity and disability burden among older adults.

**Revision:**

*Discussion*

The preponderance of women exceeds their proportion in the general older population (approximately 56.0% in 2023 [23]), likely reflecting the ‘male–female health–survival paradox’ where women live longer but with higher disability burdens [24,25].

↓

The preponderance of women exceeds their proportion in the general older population (approximately 56.0% in 2023 [23]), which may partly reflect sex differences in longevity and disability burden among older adults, as women generally live longer but experience higher disability burdens [24,25].

**Comment 33.**

The manuscript interprets the high proportion of Medical Aid beneficiaries as evidence that LTCHs function as a “social safety net.” However, Medical Aid beneficiaries may utilize LTCHs because they have fewer alternatives for long-term care, such as limited family support or financial resources, rather than because LTCHs are specifically designed to serve this role.

**Response:**

We appreciate the reviewer’s thoughtful comment. We revised the Discussion section to present a more cautious interpretation.

**Revision:**

*Discussion*

Collectively, these results suggest that LTCHs are functioning not merely as medical facilities but as a social safety net for older adults.

↓

Collectively, these findings suggest that LTCHs may play an important role in the care of socioeconomically vulnerable older adults who may have limited alternatives for long-term care.

**Comment 34.**

Although the manuscript appropriately notes that final claim diagnoses reflect managed conditions rather than underlying causes of death, it does not acknowledge that claims data lack important clinical information such as functional status, cognitive impairment, and frailty, which are critical for understanding end-of-life trajectories in LTCHs.

**Response:**

We appreciate the reviewer’s important comment. We added this point to the Limitations section.

**Revision:**

*Discussion* (Newly added)
First, this study relies on administrative claims data, which inherently lack detailed clinical information such as functional status, cognitive impairment, and frailty, all crucial for understanding end-of-life trajectories.

**Comment 35.**

The limitations section mentions caregiving costs but does not acknowledge other potentially important out-of-pocket expenses, such as uncovered medications, medical supplies, or private-duty nursing.

**Response:**

We appreciate the reviewer’s important comment. We revised the Limitations section to clarify this point.

**Revision:**

*Limitation section*

Second, as this study relied on administrative claims, data on non-covered services (e.g., caregiving costs) were unavailable, likely resulting in an underestimation of the total economic burden.

↓

Finally, our economic estimates likely underestimate the total financial burden because non-covered out-of-pocket expenses (e.g., caregiving, medical supplies) were unavailable.

**Comment 36.**

The manuscript describes the descriptive nature of the analysis as a limitation. However, this reflects an analytic choice rather than a limitation inherent to the dataset itself.

**Response:**

We appreciate the reviewer’s thoughtful comment. We respectfully note that we had originally acknowledged this in the Limitations section ("Finally, our analysis was descriptive by design…"). Nevertheless, to clarify this point, we have further expanded this paragraph.

**Revision:**

*Limitation section*

Finally, although the large nationwide dataset provided substantial analytical potential, our analysis remained primarily descriptive and did not include multivariable modelling or stratified analyses to account for potential confounding factors such as age and sex.

↓

Second, this study was designed primarily as a descriptive population-level analysis. Without multivariable modeling or stratified analyses, we could not evaluate how clinical trajectories might differ across specific demographic subgroups …

**Comment 37.**

The Conclusion states that LTCHs in Korea have become a primary setting for end-of-life care, but it does not present the key supporting figure that 68.5% of decedents died during LTCH stay.

**Response:**

We appreciate the reviewer’s helpful comment. We revised the Conclusion section to include the proportion of decedents who died during the LTCH stay.

**Revision:**

*Conclusions*

In conclusion, this decade-long study shows that LTCHs in Korea have transitioned into a primary setting for end-of-life care, managing a distinct trajectory from chronic frailty to acute deterioration.

↓

In conclusion, this decade-long study shows that LTCHs in Korea have transitioned into a primary setting for end-of-life care, with more than two-thirds of decedents (68.5%) dying during LTCH stay. The findings further suggest a distinct trajectory from chronic frailty to acute deterioration near the end of life.

**Comment 38.**

The policy recommendation in the Conclusion is somewhat vague and not sufficiently actionable.

**Response:**

We appreciate the reviewer’s helpful comment. We revised the Conclusion section to provide more concrete and actionable recommendation.

**Revision:**

*Conclusions*

Moving beyond a singular focus on rehabilitation, these models should integrate palliative care principles to ensure dignified death for the growing population of frail older adults.

↓

Given that institutions designed for sub-acute rehabilitation are effectively functioning as end-of-life care settings, policy reforms should strengthen palliative care integration, advance care planning, workforce training, and end-of-life care capacity within LTCHs to better address the complex needs of frail older adults.

**Comment 39.**

They did not measure patient-reported outcomes, family satisfaction, or any quality indicators of end-of-life care. Therefore, this claim "death with dignity" from this study is not based on evidence.

**Response:**

We appreciate the reviewer’s thoughtful comment. We revised the Conclusion section to avoid language implying conclusions beyond the scope of the available evidence.

**Revision:**

*Conclusions*

Moving beyond a singular focus on rehabilitation, these models should integrate palliative care principles to ensure dignified death for the growing population of frail older adults.

↓

Given that institutions designed for sub-acute rehabilitation are effectively functioning as end-of-life care settings, policy reforms should strengthen palliative care integration, advance care planning, workforce training, and end-of-life care capacity within LTCHs to better address the complex needs of frail older adults.

**Comment 40.**

Reference 4: DOI is incomplete

**Response:**

We appreciate the reviewer’s careful comment. We corrected the incomplete DOI in Reference 4.

**Revision:**

*References*

Boo S, Lee J, Oh H. Cost of care and pattern of medical care use in the last year of life among long-term care insurance beneficiaries in South Korea: Using national claims data. *Int J Environ Res Public Health*. 2020;17(23):9078. doi:0.3390/ijerph17239078

↓

Boo S, Lee J, Oh H. Cost of care and pattern of medical care use in the last year of life among long-term care insurance beneficiaries in South Korea: Using national claims data. *Int J Environ Res Public Health*. 2020;17(23):9078. doi:10.3390/ijerph17239078

**Comment 41.**

Reference 19: no URL or DOI. For a guideline document, provide a persistent identifier or repository link.

**Response:**

We appreciate the reviewer’s helpful comment. We revised the reference to include the official repository URL from the Health Insurance Review and Assessment Service website.

**Revision:**

*References*

Health Insurance Review and Assessment Service (Korea). *Standard Guidelines for Calculating Disease and Medical Procedure Statistics 2024*. 2024.

↓

Health Insurance Review and Assessment Service (Korea). *Standard Guidelines for Calculating Disease and Medical Procedure Statistics 2024*. 2024. Available from: https://opendata.hira.or.kr/

**Comment 42.**

Reference 26: Health Insurance Review & Assessment Service, 2025 – that's very recent (published after the study period ended). Was it available when they wrote the manuscript?

**Response:**

We appreciate the reviewer’s careful comment. Because the manuscript was finalized in early 2026, we were able to utilize the reference published in 2025.

**Comment 43.**

Several references (9, 10, 11, 13, 14, 15) are older (>5 years) and focused on small cohorts.

**Response:**

We appreciate the reviewer’s comment. Because our study analyzed a decade of longitudinal data from 2014 to 2023, we sought references to prior studies conducted during this timeframe.

**Reviewer 3**

**Comment 1.**

Three groups were constructed based on all inpatients, new admissions and deaths. As these groups were not really followed up on, it is not appropriate to call them cohorts. Suggest not using the term 'cohort' when referring to these groups.

**Response:**

We appreciate the reviewer’s thoughtful comment. As suggested, we revised the terminology throughout the manuscript to “group” instead of “cohort.”

**Revision:**

annual inpatient cohort → annual inpatient group

new admission cohort → newly admitted inpatient group

decedent cohort → decedent group

**Comment 2.**

In Table 1, how were the compound annual growth rates calculated? It appears that these have been calculated using only the numbers for the first and last years. It is more appropriate to use all available data. This could be done by calculating the slope of the linear regression.

**Response:**

We appreciate the reviewer’s comment. In the original manuscript, CAGR was used to summarize the overall change from the first to the last observed year. In the revised manuscript, we additionally performed linear regression analyses across the entire study period and added p for trend values to Table 1. The Statistical Analysis section and corresponding table footnotes were revised accordingly.

**Revision:**

*Statistical analysis*

We summarized annual counts and proportions by calendar year and calculated compound annual growth rates (CAGR) to quantify trends over the study period.

↓

We summarized annual counts and proportions by calendar year and calculated compound annual growth rates (CAGR) based on the first and last observed annual values to quantify overall temporal changes during the study period. In addition, linear regression analyses using annual data across the entire study period were performed to assess temporal trends.

*Table 1*

| **CAGR (%)** | **2.91** | **0.87** | **5.66** |
| --- | --- | --- | --- |

↓

| **CAGR (%)** | **2.91** | **0.87** | **5.66** |
| --- | --- | --- | --- |
| **p for trend** | **0.006** | **0.727** | **< 0.001** |

… CAGR was calculated using the first and last observed annual values. P for trend was obtained using linear regression analyses across annual data points. …

**Comment 3.**

The following part of the abstract (in the results section) could be misleading - "... and exhibited high sociodemographic vulnerability, characterised by a predominance of females, ...". The predominance of females reflects the sex ratio in this age group. Based on this observation, we cannot say females are more vulnerable than males.

**Response:**

We appreciate the reviewer’s thoughtful comment. We revised the sentence in the Abstract to separate the descriptive finding of female predominance from indicators of sociodemographic vulnerability.

**Revision:**

Patients typically entered LTCHs in their early 80s and exhibited high sociodemographic vulnerability, characterised by a predominance of females, Medical Aid beneficiaries and individuals with disabilities.

↓

Patients were newly admitted to LTCHs in their early 80s (median age 81 years, IQR 75–86) and were predominantly female. In addition, a substantial proportion were Medical Aid beneficiaries and individuals with disabilities, indicating high sociodemographic vulnerability.
