# Supplementary material for: Long-term care hospitals as end-of-life care settings in South Korea: A nationwide analysis of utilization patterns and clinical trajectories
Source: PLoS One. 2026 Jul 24;21(7):e0354271. doi: 10.1371/journal.pone.0354271 (PMC13399523; doi:10.1371/journal.pone.0354271)
Supplement: Attachment — Submitted filename: Response_to_reviewers_260630.docx [file pone.0354271.s006.docx]

**Point-by-point response**

**Journal Requirements**

**Comment 1.**

If the reviewer comments include a recommendation to cite specific previously published works, please review and evaluate these publications to determine whether they are relevant and should be cited. There is no requirement to cite these works unless the editor has indicated otherwise.

**Response:**

We appreciate the journal’s guidance. We reviewed the reviewer’s comments and confirmed that they did not include a recommendation to cite any specific previously published works.

**Comment 2.**

Please review the reference list to ensure that it is complete and correct. If you have cited papers that have been retracted, please include the rationale for doing so in the manuscript text, or remove these references and provide the appropriate justification.

**Response:**

We thank the editorial office for this comment. We carefully reviewed the entire reference list. Each reference was verified against its original source, and we confirmed that all bibliographic details are accurate. None of the cited references has been retracted. In addressing the reviewer’s comments, we additionally cited four references and verified each against its original source.

**Reviewer 2**

**Comment 1.**

The title is still too vague and does not reflect the main finding that LTCHs function as end-of-life settings.

**Response:**

We appreciate the reviewer’s insightful comment. We revised the title to more clearly reflect the main finding of our study regarding the role of LTCHs as end-of-life care settings.

**Revision:**

*Title*

Utilization and end-of-life trajectories in long-term care hospitals of South Korea

↓

Long-term care hospitals as end-of-life care settings in South Korea: A nationwide analysis of utilization patterns and clinical trajectories

**Comment 2.**

The abstract needs key percentages and more concrete policy recommendations.

**Response:**

We appreciate the reviewer’s valuable suggestion. We revised the Abstract to include key percentages—covering the sociodemographic profile, length of stay, cost-related variation, and the end-of-life diagnostic shift—and to provide more concrete policy recommendations.

**Revision:**

*Abstract (Results)*

Patients were newly admitted to LTCHs in their early 80s (median age 81 years, IQR 75–86) and were predominantly female. In addition, a substantial proportion were Medical Aid beneficiaries and individuals with disabilities, indicating high sociodemographic vulnerability. Decedents spent a median of 79 days (IQR 22–299) as LTCH inpatients, and 68.5% died via LTCHs. While admissions were driven by chronic geriatric syndromes, the end-of-life phase was dominated by a surge in acute infectious and systemic deterioration.

↓

Patients were newly admitted to LTCHs in their early 80s (median age 81 years, IQR 75–86) and were predominantly female (62.1%). In addition, Medical Aid beneficiaries (13.7%) and individuals with registered disabilities (28.0%) were overrepresented, indicating high sociodemographic vulnerability. Decedents spent a median of 79 days (IQR 22–299) as LTCH inpatients. While 30.1% stayed for <30 days, 22.2% remained hospitalized for ≥360 days, indicating substantial variation in length of stay. Overall, 68.5% of decedents died during LTCH stay. While admissions were driven by chronic geriatric syndromes, the end-of-life phase was dominated by a surge in acute infectious and systemic conditions (e.g., pneumonia, 10.2% to 22.3%; sepsis, 3.3% to 11.9%).

*Abstract (Conclusions)*

LTCHs in Korea have transitioned into a primary setting for end-of-life care, managing a distinct trajectory from chronic frailty to acute deterioration. Given that institutions are functioning as end-of-life care settings, policies supporting palliative care integration and end-of-life care capacity within LTCHs are needed.

↓

LTCHs in Korea have played an increasingly important role as a setting for end-of-life care, managing a distinct trajectory from chronic frailty to acute deterioration. Given that these institutions are functioning as end-of-life care settings, policy reforms should strengthen palliative care integration, advance care planning, workforce training, and end-of-life care capacity within LTCHs.

**Comment 3.**

The introduction would benefit from moving the admission-mortality paradox observation earlier to frame the study's importance.

**Response:**

We appreciate the reviewer’s insightful comment. We revised the Introduction to foreground the evolving role of LTCHs in end-of-life care and to better frame the study’s importance. We anchored this framing in published national evidence on the rising proportion of institutional deaths among older Korean adults, and presented the divergence between LTCH admissions and LTCH-associated deaths as the rationale for the present study.

**Revision:**

*Introduction*

Although previous studies on Korean LTCHs have investigated specific clinical outcomes (e.g., length of stay (LOS) or functional status), they often lacked a comprehensive evaluation of longitudinal end-of-life trajectories and diagnostic shifts.

↓

In Korea, deaths among older adults increasingly occur in hospitals and other institutional settings rather than at home [9]. Although LTCHs were established primarily for subacute and rehabilitative care, they may be playing an increasingly important role in this institutional end-of-life care. The divergence between relatively stable LTCH admissions and a disproportionate rise in LTCH-associated deaths suggests an evolving role that has not been comprehensively investigated at the national level. Although previous studies on Korean LTCHs have investigated specific clinical outcomes (e.g., length of stay (LOS) or functional status), they often lacked a comprehensive evaluation of longitudinal end-of-life trajectories and diagnostic shifts that could explain this changing role.

*Reference (Newly added)*

9. Mai TTX, Lee E, Cho H, Chang YJ. Increasing trend in hospital deaths consistent among older decedents in Korea: a population-based study using death registration database, 2001–2014. BMC Palliat Care. 2018;17(1):16.

**Comment 4.**

Methodologically, the one-year washout period limitation and the missing time-gap data due to expired access are underplayed; these affect the validity of the “near-death” interpretation more than acknowledged.

**Response:**

We appreciate the reviewer’s important comment. We revised the Methods and Limitations sections to more explicitly acknowledge the potential misclassification resulting from the one-year washout period and the uncertainty arising from the unquantified time interval between the final LTCH claim and death. To align the wording with this more cautious interpretation, we also replaced descriptions of diagnoses occurring “near death” throughout the Results and figure/table captions with the more precise “final claim before death.”

**Revision:**

*Methods*

… strictly excluding those with any prior LTCH admission history recorded in the dataset since 2014 (using a minimum one-year washout period based on available claims data).

↓

… strictly excluding those with any prior LTCH admission history recorded in the dataset since 2014 using a minimum one-year washout period based on available claims data. Because claims data prior to 2014 were unavailable, a longer washout period could not be applied.

*Discussion (Limitations)*

Because claims prior to 2014 were unavailable, some individuals classified as newly admitted may have had unobserved prior admissions.

↓

Because claims prior to 2014 were unavailable, some individuals classified as newly admitted may have had unobserved prior admissions, resulting in potential misclassification of newly admitted patients. Consequently, the time from initial LTCH admission to death may have been underestimated for these individuals, potentially limiting the accuracy of the reconstructed end-of-life trajectories. Furthermore, the interval between the final LTCH claim and death could not be quantified. Therefore, diagnoses recorded on the final claim may not accurately represent patients’ clinical conditions immediately before death, particularly among those who were discharged or received care elsewhere before death. This limitation introduces uncertainty into the timing and interpretation of the observed diagnostic shifts.

*Results and Fig 2 caption (terminology)*

Sepsis increased from 3.3% at admission to 11.9% near death … (Fig 2) diagnoses at the time of admission and near the time of death

↓

Sepsis increased from 3.3% at admission to 11.9% at the final claim … (Fig 2) diagnoses at the time of admission and at the final claim before death

*S2 Table caption (terminology)*

S2 Table. … recorded clinical conditions at admission and near death among older decedents in LTCHs.

↓

S2 Table. … recorded clinical conditions at admission and at the final claim before death among older decedents in LTCHs.

**Comment 5.**

The results on heterogeneity in length of stay and cost skew are interesting but not adequately explored; the paper does not examine what distinguishes short-stay from long-stay patients.

**Response:**

We appreciate the reviewer’s insightful comment. Because additional subgroup analyses were not feasible within the available data access period, we revised the Results and Discussion to better describe the heterogeneity in length of stay and costs, offered a plausible interpretation of the short- versus long-stay groups while making clear that it could not be confirmed, and explicitly acknowledged the absence of patient-level comparisons as a limitation.

**Revision:**

*Results*

Regarding expenditures, the median cumulative medical cost from admission to death was approximately 7.3 million Korean Won.

↓

Regarding expenditures, the median cumulative medical cost from admission to death was approximately 7.3 million Korean Won (KRW); 42.2% incurred cumulative medical costs of ≤5 million KRW, whereas 30.5% incurred costs of ≥20 million KRW.

*Discussion*

While the patients spent a median of 79 days as an LTCH inpatient, the population was heterogeneous, comprising both short-stay users likely seeking terminal care and long-term residents. The wide variation in cumulative medical costs further suggests substantial heterogeneity in end-of-life care utilization and may reflect differences in clinical complexity, care needs, and financial burden among LTCH patients.

↓

While the patients spent a median of 79 days as an LTCH inpatient, the population was heterogeneous, comprising both short-stay users and patients with prolonged institutional stays. This bimodal pattern may reflect a duality in which shorter stays plausibly represent admissions for terminal care and longer stays reflect chronic institutional residence. However, the present descriptive analysis could not determine which patient characteristics distinguished short-stay from long-stay users, and these interpretations require further investigation. The wide variation in cumulative medical costs further indicates substantial heterogeneity in resource use. Future studies should examine the patient- and facility-level factors associated with differences in LOS and cumulative medical costs.

*Discussion (Limitations)*

Without multivariable modeling or stratified analyses, we could not evaluate how clinical trajectories might differ across specific demographic subgroups, including detailed characteristics such as disability type and severity.

↓

Without multivariable modeling, stratified analyses, or patient-level comparisons, we could not evaluate how clinical trajectories might differ across demographic subgroups (e.g., disability type and severity) or identify the characteristics that distinguished short-stay from long-stay users or accounted for the wide variation in cumulative costs.

**Comment 6.**

The discussion overstates the “social safety net” narrative and could more openly acknowledge that the diagnostic shift finding aligns with existing literature. The conclusion remains somewhat strong given the limitations. A more balanced tone and clearer statement about what is truly novel would strengthen the manuscript.

**Response:**

We appreciate the reviewer’s insightful comment. We moderated the “social safety net” interpretation, added an explicit acknowledgment that the observed diagnostic shift is consistent with existing literature on the end-of-life course in older adults, clarified what is genuinely novel about our study (its nationwide, decade-long scope), and adopted a more balanced tone in the Discussion and Conclusion. For consistency, the same balanced wording was applied to the corresponding statements in the Abstract.

**Revision:**

*Discussion (social safety net)*

Furthermore, economic deprivation appears to be a key driver of institutionalization. The proportion of Medical Aid beneficiaries in our group (13.7%) was more than four times the rate in the general Korean population (approximately 3% [26]). Collectively, these findings suggest that LTCHs may play an important role in the care of socioeconomically vulnerable older adults who may have limited alternatives for long-term care.

↓

The proportion of Medical Aid beneficiaries in our group (13.7%) was more than four times the rate in the general Korean population (approximately 3% [27]). Collectively, these findings indicate that socioeconomically and physically vulnerable older adults were disproportionately represented among LTCH admissions.

*Discussion (diagnostic shift; overstatement removed and literature alignment added)*

Although admissions were largely driven by chronic multimorbidity, the period preceding death was marked by a ‘terminal cascade,’ a dramatic shift where frailty and functional decline precipitate acute infectious and respiratory deterioration.

↓

Although admissions were largely driven by chronic multimorbidity, the period preceding death was characterized by a shift toward acute infectious and respiratory conditions. Such a shift is consistent with prior studies of the end-of-life course in older adults, in which terminal decline is frequently accompanied by acute infections such as pneumonia and sepsis [28–30].

*Discussion (Strengths; statement of novelty added)*

Taken together, these features provide an integrated, nationwide characterization of LTCH utilization that links trends in admissions and mortality with cumulative LOS, medical costs, and diagnostic change from admission to the final claim before death, which to our knowledge has not previously been reported.

*Conclusions*

In conclusion, this decade-long study shows that LTCHs in Korea have transitioned into a primary setting for end-of-life care, with more than two-thirds of decedents (68.5%) dying during LTCH stay. The findings further suggest a distinct trajectory from chronic frailty to acute deterioration near the end of life. Given that institutions designed for sub-acute rehabilitation are effectively functioning as end-of-life care settings, policy reforms should strengthen palliative care integration, advance care planning, workforce training, and end-of-life care capacity within LTCHs to better address the complex needs of frail older adults.

↓

Our findings suggest that LTCHs have played an increasingly important role as end-of-life care settings in Korea, with more than two-thirds of decedents (68.5%) dying during LTCH stay. The findings also showed a shift from chronic geriatric conditions at admission toward more frequent acute infectious and systemic conditions in the final observed claims before death. Given the growing role of LTCHs in end-of-life care, policy reforms should strengthen palliative care integration, advance care planning, workforce training, and end-of-life care capacity within LTCHs to better address the complex needs of frail older adults.

*References (Newly added)*

28. Mitchell SL, Teno JM, Kiely DK, Shaffer ML, Jones RN, Prigerson HG, et al. The clinical course of advanced dementia. N Engl J Med. 2009;361(16):1529-1538.

29. Tark A, Estrada LV, Tresgallo ME, Quigley DD, Stone PW, Agarwal M. Palliative care and infection management at end of life in nursing homes: A descriptive survey. Palliat Med. 2020;34(5):580-588.

30. Quah WC, Leong CJ, Chong E, Low JA, Rafman H. Unplanned hospitalisations among subsidised nursing home residents in Singapore: Insights from a data linkage study. Ann Acad Med Singap. 2024;53(11):657-669.

*We note that Reviewer 2's comments referenced an attached file with further detail. However, the Academic Editor confirmed that there is no such file.*

**Reviewer 3**

We sincerely thank Reviewer 3 for the previous review; no further comments were raised.
